# Supplementary material for: Are carbon emissions trading and green financial instruments synergistic? -Comprehensive quantitative research based on content analysis
Source: PLoS One. 2024 Mar 7;19(3):e0298601. doi: 10.1371/journal.pone.0298601 (PMC10919601; doi:10.1371/journal.pone.0298601)
Supplement: S1 Data — (ZIP) [file pone.0298601.s001.zip › Data/地级市Z_F工作报告.zip!/║╙─╧/─╧╤⌠╩╨/└┤╫╘╣1⁄2╓┌║┼í╛┬φ┐╦╩2╛▌═°í┐_2011.docx]

各位代表:

现在我代表市人民政府向大会作工作报告请予审议并请各位政协委员和其他列席人员提出意见。

折叠编辑本段详细内容

一、去年及"十一五"工作回顾

(一)保增长调结构经济发展提速增效。预计去年全市生产总值完成1956亿元增长11.6%。财政总收入122.9亿元、增长21%其中地方财政一般预算收入69.1亿元、增长23%。工业经济快速增长。规模以上工业完成增加值634亿元增长22.4%增速居全省第二位实现利润130亿元增长66.8%创近年来最高水平。农村经济稳步发展。粮食总产达116.8亿斤连续7年创历史新高农业结构不断优化畜牧业占农业总产值的比重达36.5%农业产业化经营水平明显提升39家企业被认定为省级农业产业化重点龙头企业。第三产业快速发展。全社会消费品零售总额完成789亿元增长18.7%。实现旅游综合收入95.6亿元增长37.9%。全市金融机构各项贷款余额827.5亿元增长18.4%。民生、中信、郑州银行和中航、中信证券相继入驻南阳与天津市合作设立的南阳村镇银行及2家支行挂牌营业开创我国总行加支行设立村镇银行新模式。

(二)两轮驱动统筹推进城乡建设步伐加快。中心城区加速发展鸭河、官庄工区成立运行南阳新区获省政府批准高新区晋升为国家级高新区。中心城市基础设施投资超过30亿元是历年来投资强度最大、施工项目最多的一年。6条道路和仲景大桥建成通车62条背街小巷整治改造顺利完成光武大桥、雪枫大桥、南阳大桥加宽改造、零级橡胶坝和邕河整治试验段开工建设内河治理启动实施白河城区段确权划界实现突破。严厉打击土地违法违规行为中心城区土地收储力度加大。新投放出租车400辆、公交车100辆。实施"现代城镇体系建设工程"县城和小城镇建设步伐加快亮点纷呈。深入开展"六创一迎"城镇品位和形象明显提升。农村生产生活条件不断改善完成了100个试点村、100个示范村等新农村年度建设任务再次夺得全省农建"红旗渠精神杯"。交通建设扎实推进新建、改建干线公路360公里建设农村公路1430公里新增南阳直飞北京航班和到上海始发列车。生态建设和环境保护得到加强植树造林83.7万亩节能减排任务如期完成。

(三)集中精力抓大事重点工作进展顺利。产业集聚区建设步伐加快。全市14个产业集聚区累计完成投资435亿元、实现规模以上工业主营业务收入800亿元8个集聚区在全省位次明显前移高新技术产业集聚区跻身省创新型产业集聚区。大招商活动成效显著。与央企的战略合作进一步深化防爆与平安集团、天冠与首钢控股成功合作全年实际到位省外资金273亿元、增长一倍实际利用外资超2亿美元、增长51.2%出口总额6.47亿美元、增长50%。第七届全国农运会筹备工作全面展开。面对拆迁建设任务重、资金缺口大、工期时间紧等困难我们负重奋进只争朝夕确保了各项任务按时间节点顺利推进。主体育场、综合训练馆、游泳馆等主体工程完工新建、改造提升了15个场馆和一批接待设施。南水北调中线工程移民迁安和工程建设取得阶段性重大进展。1.1万试点移民后续生产生活保持稳定库区第一批6.08万移民实现平安、顺利、和谐搬迁第二批移民迁安工作扎实有序推进中线工程建设进展顺利。移民搬迁的难度之大、强度之高、速度之快创造了近年来的新记录受到国家领导和省委、省政府的充分肯定在中国移民史上留下了浓墨重彩的一笔!移民群众舍小家、为国家的奉献精神移民干部任劳任怨的责任意识谱写了新时期可歌可泣的壮丽篇章!

(四)谋长远打基础发展后劲不断增强。"四个带动"初见成效。实施项目带动年度城镇固定资产投资首次突破千亿完成1130亿元、增长21.6%内邓高速、南阳机场二期改扩建等省市重点项目进展顺利南阳核电等重大项目前期工作取得突破性进展全市共筛选确定"十二五"重大项目2733个、总投资14000多亿元其中进入全省重大项目库1981个、总投资8600多亿元。实施品牌带动新创建国家级品牌57个、省级品牌363个。实施创新带动争取国家重大科技项目21项获得省科技进步奖21项其中一等奖3项加强创新平台建设卧龙农业高新科技示范园区晋升为国家级荣获"中国最具创新力城市"称号。实施重点改革行动计划国企改革攻坚战取得显著成效19家改制企业焕发生机市县政府机构改革基本完成公安警务机制改革取得突破启动实施医院用药阳光集中配送医药卫生体制改革取得新成效教育、文化等领域改革继续深化积极推进"421"企业上市计划新纺公司股票实现定向增发西泵公司成功上市。实施服务带动扎实开展"企业服务行动计划"发展环境不断优化。注重科学规划的引领和提升作用聘请名院大家汇集民智高起点、高标准编制了"十二五"发展规划和现代产业规划、城市重点区域规划等一系列重要规划为南阳未来科学发展、跨越发展提供了有力保障。

(五)致力改善和保障民生和谐社会建设稳步推进。围绕解决就业、就学、就医、住房和社会保障等问题投入资金50亿元顺利完成了"十项民生工程"。城镇居民人均可支配收入、农民人均纯收入分别增长11.7%和14.9%。新增城镇就业10.4万人失业人员再就业近3万人。社会保障体系进一步健全新农保全国试点县达到5个新农合参合率达97.7%。建设经济适用房56.8万平方米新增廉租房8980套。解决农村47.2万人饮水安全问题新建户用沼气3.15万户11.25万农村贫困人口实现脱贫。抗洪抢险、救灾重建工作取得重大胜利。教育、文化、卫生、体育等各项社会事业全面发展市职教园区被确定为全省5个示范性职教园区之一。人口自然增长率控制在5.07%。。深入开展精神文明创建活动公民道德水平和文明程度不断提升。认真开展"创先争优"活动全面推广"四议两公开"工作法基层组织建设和农村基层民主得到加强。坚持依法行政行政执法水平明显提高。自觉接受人大及其常委会的监督支持政协履行职能办理省市人大代表建议和政协委员提案703件办结率100%满意率95.6%。开展"夏季亮剑"百日会战等专项行动社会治安明显好转平安建设位次大幅提升。认真实施安全发展行动计划强化食品药品安全监管扎实推进信访工作社会大局和谐稳定。

国防动员、民兵预备役和全国双拥模范城创建工作深入开展。工商、税务、司法、海关、检验检疫、人事、统计、审计、广电、新闻、民族宗教、外事侨务、对台、残疾人、气象、人防、地震等工作取得新成绩。第六次全国人口普查工作进展顺利。

一年来面对保增长与调结构的双重压力面对史无前例的移民迁安重任面对异常艰巨的农运会筹备任务面对百年不遇的洪涝灾害全市上下咬定发展不放松加快转型不懈怠深入实施关系长远的"四个带动"全力抓好事关全局的"国字号"工程办成了一批大事办妥了一批难事跻身中国城市竞争力百强在困难中保持了较快发展在拼搏中铸就了新的辉煌!实践证明我们南阳人不仅会干事、能干事而且能干成大事!2010年各项工作目标的圆满完成标志着"十一五"规划的胜利实现。过去五年是南阳发展史上极不平凡的五年。

---综合实力明显提高。按可比口径计算全市生产总值年均增长12.5%三次产业结构由"二、一、三"升级为"二、三、一"。地方财政一般预算收入、规模以上工业增加值和利润均翻一番多。全市实际利用外资、引进省外资金年均分别增长26.2%、27.8%。

---发展后劲不断增强。全社会固定资产投资五年累计完成4600亿元超出规划目标1300亿元。鸭电二期、1000千伏特高压南阳开关站等一大批重点项目相继建成。高新技术产业化步伐加快先后被确定为新能源国家高技术产业基地、国家光电高新技术产业化基地、省生物产业高技术产业基地。

---城乡面貌大为改观。中心城区建成区面积由70平方公里增至100平方公里人口增至100万荣获中国优秀旅游城市、国家园林城市等称号。中国·南阳伏牛山世界地质公园申报成功。县城和小城镇建设迈出重大步伐。全市城镇化率达到38.5%提高8.5个百分点。宁西铁路建成通车南阳机场新航站楼建成启用公路通车总里程增长3.7倍高速公路通车里程达553公里、居全省第一被交通运输部确定为国家级公路运输枢纽城市新建改建农村公路1.5万公里"村村通"工程全面完成。解决了160余万农村居民饮水安全问题新增农村沼气用户33万户累计造林422万亩。

---社会事业全面进步。教育"两基"工作通过国检城乡免费义务教育全面实现各级各类教育协调发展。文化、体育事业日益繁荣。科技对经济增长的贡献率由42.7%提高至49%荣获"全国科技进步先进市"称号。医疗卫生服务体系逐步健全被评为"全国农村中医工作先进市"。计划生育工作机制创新力度加大被确定为全国首批计划生育综合改革示范市之一。

---人民生活持续改善。城镇居民人均可支配收入、农民人均纯收入年均分别增长13.6%、13.3%。城乡居民储蓄存款余额年均增长15.3%五年翻一番多。坚持每年办好十项民生工程新增城镇就业50万人农村富余劳动力转移就业76万人各类社会保障覆盖面持续扩大、标准不断提高。52万贫困人口实现脱贫。各位代表"十一五"时期的发展成就是在国际金融危机严重冲击、宏观环境复杂多变、重大自然灾害频发的情况下取得的。这是省委、省政府和市委正确领导的结果是市人大、市政协积极支持的结果是全市人民共同奋斗的结果。在此我代表市人民政府向全市广大干部群众向各位人大代表、政协委员和各民主党派、工商联、无党派人士向离退休老同志向驻宛部队、武警官兵向中央、省驻宛单位向所有关心支持南阳发展的各界朋友表示崇高的敬意和衷心的感谢!

五年的成绩来之不易奋斗的启示弥足珍贵。我们深切体会到推进政府工作必须着眼全局和长远自觉把南阳放在全省全国发展的大格局中来谋划多做打基础、利长远、增后劲的事情必须注重统筹和运作在经济社会发展上统筹兼顾强化运作、有效运作抢抓机遇谋发展突出重点攻难点推动整体工作全面提升必须突出项目和产业坚持以项目建设统揽经济社会发展全局着力培育壮大战略支撑产业和新兴产业以项目建设和产业振兴支撑发展必须抓好平台和载体积极实施"四个带动"着力构建"一个载体、三个体系"深入开展"六创一迎"办好节会充分发挥这些平台、载体的带动作用必须坚持求实求效加强效能建设优化政务环境强化督查落实注重效率和效益不断提高政府执行力和公信力必须关注民生民心把发展经济与改善民生结合起来尽心尽力为群众办实事、办好事让改革发展成果更多地惠及人民。同时我们也清醒地看到南阳人口多、基础弱、大而不强的基本市情还没有根本改变。当前发展中还存在不少困难和问题经济发展速度不快主要经济指标人均水平在全省仍处于中等或靠后位次经济结构不优农业基础设施薄弱工业实力不强三产发展滞后城镇化水平较低中心城市首位度不高体制机制性矛盾仍然突出发展动力不足、活力不强基本公共服务薄弱社会事业历史欠账较多尤其是教育和医疗资源严重不足、布局不合理改善民生的任务十分艰巨政府自身建设和管理需要加强一些部门办事效率不高一些工作人员的思想观念、能力素质和工作作风还不适应新形势的需要。对此我们一定高度重视认真解决。

二、"十二五"时期的发展思路和奋斗目标

"十二五"时期是我市全面发力、加速转型、跨越发展的重要战略机遇期。综观全局当前及今后一个时期国际、国内产业梯度转移的趋势进一步增强国家进一步扩大内需、促进中部崛起、加大转移支付力度中原经济区已正式上升到国家战略层面我市被省委、省政府确定为中原经济区主体区和连南启西、对接周边的先锋区尤其令人鼓舞的是省政府最近专题出台了《关于支持南阳经济社会加快发展的若干意见》在政策、项目、资金等方面予以大力支持这些都为我市加速发展提供了前所未有的重大机遇我们必须紧紧抓住争取有更大作为。审视南阳我市正处于工业化、城镇化加速推进期经济发展的内生机制初步形成产业技术、区位交通、人文自然资源等优势日益凸显南阳新区、国家级高新区建设拉开帷幕南水北调中线工程、南阳核电、南阳粮食主产区建设等重大工程陆续上马加速发展的基础更为坚实。我们完全有条件、有信心在新的起点上实现更快速度、更大规模、更高水平的发展。

根据市委"十二五"规划建议今后五年我市经济社会发展的指导思想是深入贯彻落实科学发展观以科学发展为主题以加快转变经济发展方式为主线以富民强市为中心任务坚持"四个重在"实践要领深入实施"四个带动"大力推进"一个载体、三个体系"建设加快新型工业化、城镇化和农业现代化进程着力改善民生促进社会和谐全力打造豫鄂陕省际区域性中心城市和生态宜居城市构建中原经济区重要区域增长极为全面建设小康社会打下坚实基础。

展望"十二五"我市经济社会发展的愿景和目标是

---综合实力迈上新台阶。按照"主要经济指标年均增速高于全省平均水平、人均指标力争达到或接近全省平均水平"的要求"十二五"时期全市生产总值年均增长11%以上地方财政一般预算收入年均增长13%全社会固定资产投资五年累计突破万亿元大关经济总量占全省的比重进一步提高综合经济实力、区域竞争力显著增强成为中原经济区重要的区域增长极。

---转型发展迈出新步伐。产业结构和生产力布局得到优化科技进步对经济增长的贡献率达到55%现代产业体系、自主创新体系基本确立形成8个销售收入超500亿元的产业、5个销售收入超300亿元的产业集聚区、10家销售收入超100亿元的骨干企业建成全国重要的新能源、光电高新技术特色产业基地和重大装备制造基地粮食综合生产能力突破120亿斤农业现代化水平明显提升建成全省现代农业示范区。

---城乡建设呈现新面貌。中心城区建成区面积超过130平方公里、人口达到150万左右经济实力和辐射带动能力明显增强成为豫鄂陕毗邻地区具有较大影响力的省际区域性中心城市县域经济形成各具特色的主导产业和发展模式绝大部分县市进入全省中上游水平力争邓州城区人口达到45万、唐河城区人口达到35万其他县城人口超过或接近20万农村生产生活条件明显改善城乡统筹发展格局初步形成。南阳机场年旅客吞吐量突破50万人次力争郑渝高铁、宁西铁路复线、运十铁路南阳段建成投运所有县城20分钟以内上高速所有干线公路达到二级以上标准县乡道路基本达到三级以上标准成为中部地区新的综合交通枢纽。

---生态文明形成新优势。资源节约集约利用水平明显提高节能减排完成省定目标森林覆盖率达到40%以上南水北调中线工程南阳段全面建成经济社会发展与生态环境保护有机统一人与自然和谐相处城乡人居环境进一步改善建成资源利用合理、经济效益显著、生态环境良好的全省高效生态经济示范区成为国内外知名的生态文化旅游胜地和休闲养生之都。

---改革开放实现新突破。政府职能转变、重点领域和关键环节改革进一步深化体制机制创新迈出重大步伐对外合作领域和空间不断拓展外贸进出口总额和实际利用外资实现翻番形成全方位、多层次、宽领域的对外开放新格局成为充满活力和吸引力的内陆开放高地。

---人民生活水平得到新提高。城镇居民人均可支配收入和农民人均纯收入分别达到2.39万元、8600元以上接近全省平均水平各级各类教育快速发展医疗卫生服务能力大幅提高"上学难"、"看病难"问题得到有效缓解就业渠道更加畅通就业岗位更加丰富就业服务更加健全低收入群体住房保障问题明显改善广覆盖、多层次、可持续的社会保障体系基本建立社会更加安全稳定和谐人民生活更加幸福美好。

围绕实现上述奋斗目标我们要深入实施"四个带动"激活两个主体坚持"两轮驱动"、"三化"协调推进实施"一极两轴三区"战略布局加快构建十大战略支撑体系，即充分激活经济社会发展中各级政府这一责任主体和各类企业这一市场主体形成振兴南阳的强大合力。坚持中心城市和县域经济"两轮驱动"以新型城镇化为支撑、以新型工业化为主导、以推进农业现代化为基础奋力走出一条不以牺牲农业和粮食、生态和环境为代价的"三化"协调科学发展的路子努力破解"钱从哪里来、人往哪里去、粮食怎么保、民生怎么办"四道难题。优化战略布局强化"一极"将中心城市打造成为市域经济发展的核心增长极提升"两轴"依托承东启西的宁西铁路、312国道及沪陕高速连南接北的焦枝铁路、许南襄高速以及规划建设的郑渝高铁构建两条十字交叉的市域经济隆起带统筹推进产业集聚区、粮食主产区、生态安全功能区协调发展。构建十大战略支撑体系即着力构建具有竞争力的现代产业支撑体系、统筹城乡的新型城镇化支撑体系、现代化的基础设施支撑体系、引领发展的区域自主创新支撑体系、充满活力的体制机制支撑体系、内外互动的开放型经济支撑体系、高素质的人力资源开发支撑体系、独具特色的文化支撑体系、可持续发展的资源环境支撑体系、以人为本的和谐社会支撑体系。

《南阳市国民经济与社会发展第十二个五年规划纲要(草案)》及说明已印发大会请一并审议。

各位代表!展望未来机遇难得前景喜人，挑战严峻任务艰巨。今后五年我们面临加快发展和加快转型的双重任务破解结构性矛盾和体制机制性矛盾两大难题培育中原经济区重要区域增长极和区域竞争日趋激烈的双重挑战。同时还要完成服务保障南水北调中线工程、筹办第七届全国农运会和保证粮食稳产高产等国家重大任务。我们一定要保持清醒头脑以强烈的责任感和拼搏精神直面挑战奋发有为决不辜负党和人民的重托!我们坚信有省委、省政府和市委的坚强领导有全市1100万人民的共同奋斗任何艰难险阻都挡不住南阳跨越发展的坚定步伐我市"十二五"宏伟蓝图一定要实现也一定能够实现!

三、2011年工作安排

今年是"十二五"的开局之年也是建设中原经济区的起步之年做好今年工作意义重大。今年经济社会发展的主要预期目标是生产总值增长11%地方财政一般预算收入增长12%全社会固定资产投资增长20%社会消费品零售总额增长16%居民消费价格涨幅控制在4%左右城镇登记失业率控制在4.5%以内人口自然增长率控制在6.5%。以内城乡居民收入较快增长节能减排完成省下达目标。

实现上述目标今年政府工作要把握好以下几点一是把加快发展作为第一要务把富民强市作为中心任务紧扣科学发展主题贯穿加快转型主线努力实现经济发展速度质量效益、强市与富民的有机统一。二是突出重点把农运会筹备、南水北调移民迁安及工程建设、"一个载体、三个体系"建设、南阳新区和鸭河、官庄工区建设、招商引资作为今年工作的重中之重来抓全力推进务求突破。三是坚持把保障和改善民生作为各项工作的出发点和落脚点让人民群众享受到更多改革发展的成果。四是坚持把"四个带动"作为推动经济社会发展的总抓手切实抓紧抓好带动整体工作快速提升。

今年着重抓好以下十个方面工作

(一)加快转型优化结构提升工业竞争力。实施工业强市战略大力推进新型工业化。一是加快战略支撑产业和新兴产业规模化、高端化进程。围绕规划引领、龙头带动、项目支撑、集群发展、规模扩张的思路按照一个产业编制一个发展规划、培育1~2个龙头企业、带动一批配套协作企业、实施一批项目、组建一个专门班子、出台一套扶持政策的要求加速做大做强油碱化工、装备制造、电力能源、冶金建材、纺织服装、食品六大战略支撑产业和新能源、光电、新材料三大战略新兴产业力争一年打基础、三年上台阶、五年成规模。推进油碱化工产业转型升级。依托河南油田、中源化学等骨干企业重点抓好蜡产品、油页岩加工、碱硝化工等项目建设做大做强油碱化工产业。加快发展壮大装备制造产业。依托防爆、二机石油等龙头企业发挥我市在石油钻采、防爆电机、专用汽车及零部件等领域形成的技术优势扩大大型成套设备、工程机械、核级电机、深海钻机等优势产品规模打造全省重要的特色装备制造业基地。积极发展电力能源产业。大力支持特高压扩建工程建设推进南阳核电、天池抽水蓄能电站、宛西电厂等重大项目工作进度争取早日开工建设。推进冶金建材产业结构调整和战略重组。依托龙成、淅铝、中联、天瑞水泥等重点企业延伸钢铁、有色金属和建材产业链条重点实施淅铝超薄精箔、龙成热处理、南召碳酸钙产业开发等项目打造特种钢、PS版、金银制品和新型建材等生产基地。大力发展纺织服装产业。依托新纺、南纺、雪阳等龙头企业着力开发高档精梳纱、多种纤维混纺纱、面料、服装等产品。壮大提升食品产业。依托娃哈哈、龙大牧原、科尔沁牛业、三色鸽乳业、雨润禽业、赊店酒业、三源食品等龙头企业着力引进战略合作者延伸产业链条提升产品附加值实现产业转型升级。积极培育新能源产业。以新能源国家高技术产业基地建设为载体依托天冠、迅天宇等龙头企业着力培育生物质能源、多晶硅太阳能光伏、聚光太阳能发电装备等三大产业链推动迅天宇4000吨多晶硅、天冠沼气发电等项目建设打造全国一流的新能源研发制造基地。加快发展光电产业。用好国家光电高新技术产业化基地和省光电产业基地两块招牌依托中光学、首控光电、乐凯华光、社旗森霸等骨干企业加强与富士康等国内外大型企业的战略合作培育光电显示、光电信息记录材料和LED半导体照明三大产业链创建具有较强竞争力的光电产业基地。发展振兴新材料产业。依托天冠、西保、中南钻石等骨干企业着力培育二氧化碳全降解塑料、功能性冶金保护材料、超硬材料三大产业链。二是加快推进产业集聚区建设。开展"产业集聚区建设提速增效年"活动力争今年全市产业集聚区完成总投资超500亿元规模以上工业实现主营业务收入超1000亿元争取新能源、高新、邓州产业集聚区跻身"河南省新型工业化产业示范基地"西峡和高新产业集聚区进入全省30强。三是积极培育大型干企业和优势企业集群。继续抓好工业企业"双百"工程着力培育百亿企业鼓励河南油田、龙成进一步发展壮大支持天冠、淅铝、西保、中源化学、二机石油、中光学、防爆等骨干企业向主营业务收入达到百亿元的目标挺进。积极引导上下游企业建立战略联盟。继续做好十大纳税工业企业和十大纳税高增长工业企业评定工作激励企业加速发展壮大。大力实施"421"企业上市计划争取牧原股份、中南钻石、淅减等企业上市或通过发行审核新纺、西保、宛西、龙成发行企业债券。同时重视支持中小企业发展。做好中小企业产权交易市场试点工作。积极发展担保机构和小额贷款公司有效缓解中小企业融资难题。

(二)巩固加强"三农"工作。认真落实强农惠农政策加大"三农"投入推动"三农"工作再上新台阶。加强农业基础设施建设。抓住中央加快水利建设的重大机遇积极争取中小水库除险加固、中小河流治理、灌区节水改造和续建配套、水土保持等项目资金加快水利工程建设进一步改善我市农业生产条件。开工建设桐柏石步河水库抓好鸭河口水库和22家小一型水库除险加固工程。完成中低产田改造17万亩建设高标准农田8万亩新增有效灌溉面积10万亩、除涝面积10万亩、节水灌溉面积15万亩。大力发展现代农业。启动实施现代农业产业发展总体规划大力推进粮食主产区建设开展高产创建活动确保每个县市区建成3~5个万亩高产创建示范区力争夺取粮食丰收。积极发展高效特色农业抓好十大特色农产品基地建设。大力发展设施农业。统筹推进"菜篮子"工程建设新发展蔬菜2万亩其中设施蔬菜2000亩。大力发展畜牧业积极培育肉牛、生猪、奶牛、肉鸡四大优势产业打造南阳黄牛、牧原生猪等畜牧品牌力争全市肉蛋奶产品总量增长8%。加快农业产业化进程以引进、培育农业产业化龙头企业为主攻方向加大招商引资力度在粮、棉、油、牧、林业、水利、农机等方面面向全国引进一批大型产业化龙头企业带动南阳由传统农业向现代农业转变。加快建立现代农业科技示范体系。大力推进农业标准化生产。全面落实农产品市场准入制度。落实好农机具购置补贴政策大力实施薄弱环节机械化水平提升行动计划。搞好新农村建设。建成100个市级示范村、100个市级试点村完成26个省级示范村、1298个市级村容村貌整治村年度建设任务。改造农村公路2000公里、危桥4000延米。新解决25万农民饮水安全问题。新增农村沼气用户2万户建设农村沼气服务网点150个。新建村邮站500个。实施新一轮农村电网改造升级工程。对100个贫困村实施整村推进扶贫解决10万贫困人口的脱贫问题。

(三)大力发展服务业。以重大项目、重点企业、品牌培育、集聚区建设为重点通过创新理念、政策扶持、优化环境等措施推进服务业加速发展。规范提升传统服务业。以合理布局、规范经营、改造升级为重点推动餐饮住宿、商贸流通等传统服务业健康发展。以筹办农运会为契机提升酒店业接待水平。扎实推进"放心早餐"工程。切实抓好中心城区35处拟建菜市场的规划设计工作重点抓好7处拟建菜市场的规划选址、建设和6处已建菜市场的改造提升工作。贯彻落实家电、摩托车下乡和家电以旧换新政策全面提升"新网工程"建设水平。积极发展现代服务业。大力发展现代物流业加快各类物流园区规划建设积极推进物流企业整合和战略重组推动工商企业物流业务实行外包加快发展第三方物流。支持金融机构在南阳设立分支机构完善金融服务体系启动金融街规划建设优化金融生态环境推动金融业快速发展。加快特色文化旅游产业发展抓好伏牛山世界地质公园核心区、卧龙岗文化旅游产业集聚区、镇平玉文化改革发展试验区、社旗赊店商埠文化产业园区建设着力打造武侯祠、恐龙遗迹园、宝天曼、丹江渠首、桐柏淮源等五大龙头景区大力推进独山风景区建设培育一批精品旅游线路带动文化旅游产业快速发展。加快实施张仲景医药创新工程培育壮大集医疗、保健、教育、科研、产业、文化为一体的中医药创新体系强力推进"中医药都"建设。大力发展科技研发、技术交易、信息咨询、知识产权认证等科技服务和投资咨询、资产评估、法律、会计、职业中介等专业服务。积极推进"三网"融合加快3G网络建设。进一步抓好商业示范社区创建活动培育一批省级、国家级商业示范社区。认真贯彻落实国家房地产调控政策促进房地产市场健康发展。

(四)加快新型城镇化进程。把城镇化作为带动"三化"协调发展的着力点坚持走集约高效、生态宜居、城乡统筹、协调发展的新型城镇化道路全面实施城乡建设三年大提升行动计划力争城镇化率达到40%。加快城镇建设发展步伐。启动实施中心城区振兴工程突出项目支撑强力推进产业结构优化升级、综合交通体系、能源基础产业、社会事业等领域重大项目建设进一步增强中心城区的发展后劲和辐射带动能力。以新区、工区和产业集聚区为重点拓展中心城市发展空间加速壮大城市经济。组建南阳新区管理机构完成总体发展规划、控制性详规、核心起步区城市设计的编制工作搞好南阳新区土地和空间控制加快基础设施建设步伐加大项目引进、建设力度为《南阳新区建设总体方案》全面实施奠定坚实基础。进一步理顺鸭河、官庄工区管理体制健全机构充实人员发挥职能加快规划建设步伐力求取得突破性进展。以农运会场馆及配套设施建设为重点高标准推进东北分区建设。加大城中村和老城区开发改造力度。抓好中心城区3座大桥续建改造、23条道路改扩建。力争年底前基本完成中心城区背街小巷改造任务。抓好中心城区独山水厂、污水处理厂二期、白河南污水处理厂、污泥处置工程和垃圾处理厂二期工程建设。加快全市天然气长输管线和城区燃气输配系统建设争取西气东输二线南阳段支线6月底前建成送气。加快县城和小城镇发展。依托县域产业集聚区搞好县城规划推进基础设施建设完善城市功能加快培育一批宜居宜业的中等城市。以"争星创强"活动为载体以突出特色、挖掘内涵、提升品质为重点培育壮大50强镇突出抓好主要交通线、产业带上的小城镇建设精心打造一批人口超5万的中心镇。提升城镇规划和管理水平。统筹城镇发展各项规划加快建立起与城镇化进程相适应的基础设施、公益事业、公共交通、市场等规划体系。加强对规划实施的监督严厉打击违法建设行为。加强城市景观建设重视建筑风格塑造创建优美宜居城市。加强城市管理建立城市精细化管理长效机制开展社区达标晋星活动狠抓城市交通秩序和主要道路综合整治巩固经营秩序和小广告治理成果。全面实施市容环境和园林绿化提升行动计划启动城市数字化管理系统建设。坚持公交优先完成中心城区公交发展规划优化线路、增加班次方便市民出行。实施中心城区地下空间开发利用规划。把节地、节水、节材、节能落实到城市规划建设管理的全过程积极推进节约型城市建设。加快人口向城镇转移。以改革的精神和更大的气魄积极推动农村人口加速向城镇转移。加快户籍制度改革制定实施促进农民到城市落户的政策。着力解决好进城务工人员的就业、安居、子女入学、社会保障四个关键问题逐步使进城落户农民享有与市民平等的待遇。积极稳妥地推进城市新区、产业集聚区和城乡接合部新型农村社区建设带动城乡一体化发展。构建综合交通体系。抓好南阳机场二期改扩建工程积极争取开通新航线。配合做好宁西铁路复线和火车站改建工程相关工作。力争开工建设郑渝高铁南阳段。加快内邓高速建设进度争取三淅高速、武西高速南阳段开工建设。大力实施312国道部分界段一级路改造工程。开工建设南阳汽车北站等一批运输场站。

(五)全力做好第七届全国农运会筹备工作。筹办农运会是我市加快发展、促进转型、扩大开放的重大平台也是推进城市发展、繁荣体育事业、提高城乡文明程度的重大机遇。今年是农运会筹备的关键之年、决战之年做好今年各项筹备工作至关重要。一是广泛动员上下联动。举办农运会重在筹备、重在过程要把农运会筹备与完善城市功能、扩大对外开放、建设美好家园、提高全民素质和城乡文明程度结合起来最大限度地发挥农运会的辐射、带动和提升功能。从现在起全市上下各级各部门、社会各界人士都要积极行动起来参与、支持、服务农运会筹备工作自觉履行好"人人都是东道主我为农运做贡献"的光荣使命营造"办好农运会热情迎嘉宾"的浓厚氛围。二是加快农运会硬件建设进度。年底前完成8个新建体育场馆(场地)、7个改建场馆和新建、改造宾馆建设任务完成水、电、路、网络等基础设施和智能化工程建设确保各项硬件建设任务大头落地。三是大力推进农运会软件建设。搞好农运会资源开发和开闭幕式策划工作认真组织开展在全省全国有较大影响的主题宣传活动加强接待服务人员培训树立南阳开放文明的新形象。四是深入开展"六创一迎"活动。坚持市、县、乡、村四级联动全面推动创迎工作提速加力深入开展群众性精神文明创建活动促进城乡面貌、全民素质和城乡文明程度的全面提升。五是积极争取上级支持。抓住农运会筹备这一重大机遇用足用活相关政策筛选、策划、包装一批重大项目积极争取国家和省的支持。

(六)打好南水北调移民迁安和工程建设攻坚战。南水北调中线工程建设对于作为渠首、水源所在地和干线经过地的南阳必将产生重大而深远的影响同时也为我市转型发展、跨越发展带来了十分宝贵的发展机遇。我们一定要高度重视深入研究全力以赴为工程建设作出应有贡献在服务保障全局中更多更好地造福南阳人民。扎实做好移民迁安和后续发展工作。今年是南水北调移民迁安攻坚之年、决胜之年要坚定信心狠抓落实确保按时间节点完成任务。高标准推进第二批移民新村建设5月20日前达到搬迁入住条件8月底前完成8.6万移民搬迁安置。探索移民后期稳定发展路子全面落实各项移民帮扶政策确保移民"搬得出、稳得住、快发展、早致富"。切实搞好工程建设协调服务。今年南水北调中线工程主干渠将全面掀起建设高潮要积极做好干线工程土地征用和施工环境维护等工作确保工程建设顺利实施。加强库区生态建设和污染防治确保水质安全。

(七)深入推进科教兴宛和人才强市战略。积极推进自主创新体系建设。加大科技投入力度建立健全以企业为主体、市场为导向、产学研相结合的自主创新体系大力推进科技成果转化加快高新技术产业化步伐增强自主创新能力提高区域发展核心竞争力。加强科研基础条件建设开展重大科研项目攻关努力在新能源、光电、新材料、装备制造等领域争取一批国家、省重大科技项目创建一批国家级重点实验室、高新技术企业和省级工程技术研究中心、创新型产业集聚区。制定实施南阳市专利奖励办法。争创国家科技进步示范市和国家知识产权示范市。进一步提升高新区、新能源国家高技术产业基地和卧龙农业高新科技示范园区建设水平。实施教育优先发展战略。加大投入力度强力推进学前教育、城乡义务教育和高中阶段教育均衡发展。加快南阳中心城区和县城中小学建设和布局调整步伐。今年中心城区投入1.5亿元新建、改扩建10所以上中小学。实施"名师、名校长、名班主任"工程加大教师补进力度合理安排城乡教师交流全面提高教育质量。强力推进职教攻坚计划加快职教园区一期规划建设步伐启动工业学校、高级技工学校入驻园区工作加快南阳职业学院和镇平玉雕职业技术学院筹建进度。重视加强高等教育培育壮大一批特色专业学科积极推动南阳医专升本和河南省经济管理学校、南阳农校、南阳幼师升专工作。大力发展民办教育。强化人才支撑。重视培养企业高层管理人员努力建设一支具有战略思维、懂经营、会管理的企业家队伍。加强专业技术人才队伍建设畅通人才引进"绿色通道"加大高层次紧缺人才引进力度实施海外人才引进"百人计划"。推进创业人才"星火工程"培育创业人才队伍。加强企业博士后科研工作站和研发基地建设加大对科研院所、高等院校、文化、医疗机构等人才载体的扶持力度为吸引、留住、用好人才创造有利条件。

(八)强力推进改革开放。深入开展大招商活动。进一步强化大招商的战略地位围绕产业培育和功能提升积极与重大客商和战略合作伙伴开展创新型招商活动不断提高大招商、招大商的成效。精心筹办南阳赴环渤海、长三角等地区的大型招商活动深入开展以高新区、南阳新区、鸭河工区、官庄工区和产业集聚区为载体的专题招商活动积极参加省内外重大招商活动努力办好第九届玉雕节和第十届张仲景医药科技文化节。深化与中国兵工、中国兵装、首钢控股、中国平安等央企的战略合作抓好亚太科技、富士康、郑州日产特种车辆、东风新能源汽车等一批重大合作项目建设。建立招商引资长效工作机制狠抓跟踪督查争取项目早落地、早投产。加快南阳机场航空一类开放口岸和南阳市B类口岸作业区建设争取设立南阳出口加工区。深入推进各项改革。加快市属企业脱钩改制步伐。深化国有企业改革完善国资监管运营机制。完成市县政府机构改革评估工作积极稳妥地推进事业单位分类改革和公安警务机制改革。进一步理顺市区管理体制建立财权事权一致、充满活力、运转高效的城区管理体制。启动研究职能弱化、无实质教学任务的市属培训机构资源整合与盘活办法。完成集体林权制度改革主体任务实施配套改革。深化财政体制改革强化财源建设积极开展村级公益事业"一事一议"财政奖补工作。推进投融资体制改革增强融资能力。同时认真落实收入分配、金融、价格、教育、文化、水利等方面的改革措施。

(九)加强生态建设、环境保护和资源节约。加快林业生态市建设。坚持山区生态林、通道绿化、平原农田林网、环城和村镇绿化并重加快造林绿化步伐完成造林55万亩。加大环境保护力度。以创建国家环保模范城市为契机积极争取国家大型环保项目落户南阳大力推进唐白河、淮河流域和南水北调中线工程水源地等重点流域、区域水污染防治控制电力、钢铁、造纸、印染等重点行业主要污染物排放总量加快重点污染企业搬迁步伐全面推进农村环境综合整治。强力推进节能降耗。大力淘汰落后产能在冶金、建材、化工等行业实施高耗能设备更新改造建设批重大节能项目。加快节能服务体系建设加大节能新技术、新工艺、新产品开发推广应用力度。推进资源集约节约利用。做好循环经济试点工作。积极推进节水技术改造。坚持最严格的耕地保护制度和集约节约用地制度建立健全土地整治、集约节约用地和监督管理机制保障发展用地需求。大力开展农用地和村庄整治稳妥推进城乡建设用地增减挂钩积极探索农村宅基地有偿退出途径加大存量建设用地盘活力度提高土地资源保障能力。推动矿产资源整合提高矿产资源开发利用水平。

(十)切实保障和改善民生促进社会和谐稳定。坚持以人为本、执政为民实施好"十项民生工程"让人民群众得到更多实惠。大力推进就业促进计划和社会保障工作。实施更加积极的就业政策新增城镇就业9万人以上下岗失业人员再就业2.5万人以上其中"4050"等就业困难人员1万人"零就业家庭"动态归零新增农村劳动力转移就业8万人以上全年发放小额担保贷款2亿元以上。完善落实被征地农民就业和社会保障办法。提高企业退休人员基本养老待遇。强力推进社会保险扩面征缴清欠工作新增城镇企业职工基本养老保险1.2万人城镇居民基本医疗保险稳定在75万人以上。实现失业保险市级统筹。巩固完善新农合制度。抓好新型农村养老保险试点工作5个试点县力争参保人数达到165万人以上。扎实做好社会救助工作提高城乡低保、农村五保供养水平。重视帮扶救助"空巢"困难老人。积极推进企业职工工资集体协商制度。大规模实施保障性安居工程开工建设各类保障性住房90万平方米。加大住房公积金归集力度发挥住房公积金制度的保障作用。加快文化强市建设增强南阳软实力。实施艺术精品战略繁荣文艺创作。加快文化重点项目建设完成南阳广电中心建设任务开工建设南阳报业大厦。完善公共文化服务体系推进市县图书馆、文化馆、博物馆、档案馆和乡镇文化站、农村文化大院、广播电视村村通等文化惠民工程建设。加强国家历史文化名城建设搞好文物保护和合理利用。全面完成有线电视数字化整体平移。大力发展卫生、体育等社会事业。

深化医药卫生体制改革加快推进基本医疗保障制度建设建立基本药物制度改进社区卫生服务。健全基层医疗卫生服务体系促进城乡基本公共卫生服务均等化。推进公立医院改革试点。深入开展争创食品药品安全监管示范县、优秀城市活动保障食品药品安全。加快全民健身试点城市和国家休闲体育产业基地建设步伐完成600个村、50个社区全民健身工程。统筹做好人口计生工作稳定低生育水平提高出生人口素质优化人口结构积极应对人口老龄化。维护社会安全稳定。加强和改进信访工作不断探索以群众工作统揽信访工作的新途径推行重大决策信访风险评估引导群众以理性合法途径表达诉求。启动"安全南阳"创建工作加大责任追究力度坚决遏制各类重特大事故发生。搞好地震安全工程建设。加快推进社会治安视频监控系统建设加强社会治安综合治理保持打击各类犯罪行为的高压态势进一步提高人民群众的安全感。

同时继续加强国防动员、国防教育和人民防空工作重视支持驻宛解放军、武警部队和民兵预备役工作全面落实优抚安置政策积极创建全国双拥模范城。做好村委会换届工作。深入推行"四议两公开"工作法。支持工商联、工会、共青团、妇联、科协、文联、企联等发挥桥梁纽带作用。认真做好工商、统计、审计、民族宗教、外事侨务、残疾人、气象、地方志、档案等工作。

四、深化"四个带动"开创政府工作新局面

新的形势和任务对政府自身建设提出了新的更高的要求。我们要进一步增强忧患意识、责任意识、为民意识继续以"四个带动"为总抓手坚持依法行政从严治政强化运作求实求效推动全市经济社会科学发展、跨越发展。

(一)深入实施"四个带动"。按照"巩固、深化、创新、提高"的要求强力组织"四个带动"新突破。一是强化项目带动。健全完善项目工作机制统筹推进经济、文化、社会等领域项目建设今年全市实施140个以上投资超亿元的重大项目带动全市城镇固定资产投资完成1400亿元以上。市级重点抓好60个左右重大产业升级项目、20个重大城市建设项目、10个重大能源交通项目、10个左右重大项目前期工作。二是提升品牌带动。实施质量兴市战略和标准化战略强化品牌激励、培育发展机制建立国际、国家、省、市四级品牌梯队集中培育一批优势明显的企业品牌、产品品牌、工作品牌全年争创国家级品牌20个、省级品牌120个促进产品质量、工作标准和发展层次全面提升。三是深化创新带动。积极推进科技创新、体制机制创新和管理创新争取创建省级以上研发平台10家着力培育一批体制机制创新和管理创新典型激发各领域的创新创造活力推进创新型南阳建设。四是推进服务带动。进一步清理行政审批项目优化流程、简化程序、缩短时限完成以行政审批为重点的全市统一的行政效能电子监察系统建设研究制定绩效评估指标体系及绩效考核办法开展机关效能提升行动大力优化发展环境。

(二)强力推进依法行政。深入推进依法治市工作扎实开展法治南阳创建活动。全面贯彻依法行政实施纲要严格依照法定权限和程序履行职责。加强政府法制建设完善行政决策机制出台实施《重大行政决策规则》坚持依法决策、民主决策、科学决策。规范行政执法行为落实行政执法责任制和责任追究制。积极做好行政复议工作。自觉接受人大及其常委会的法律监督、工作监督和人民政协的民主监督切实办好人大代表建议和政协委员提案。认真听取各民主党派、社会团体和无党派人士的意见和建议。主动接受新闻舆论监督和社会公众监督。进一步扩大政务公开范围及时把与人民群众利益相关的各类事项向社会公开。

(三)坚持从严治政。深入推进"两转两提"加强政府运行管理塑造良好政风树立高效廉洁的政府形象。一是严肃政纪。修订完善并严格执行政府规则及各项工作制度强化制度约束严肃政务纪律确保令行禁止、政令畅通。二是勤政敬业。在政府系统扎实开展"创先争优"活动树立榜样拉高标杆建立激励干事创业的工作机制营造想干事、多干事、干成事的良好环境。三是从严治"奢"。严格财经纪律加强支出管理全年因公出国(境)经费、车辆购置及运行费、公务接待费等支出预算零增长制止铺张浪费降低行政成本。四是廉洁从政。认真落实领导干部廉洁自律各项规定严格执行廉政建设责任制以制度建设和权力监督为核心进一步建立健全政府系统惩治和预防腐败体系严厉查办涉农资金、环境保护、征地拆迁、重点工程建设、土地出让等方面的案件坚决纠正损害群众利益的突出问题以反腐纠风的实际成效取信于民。

(四)强化运作务求实效。强化运作意识创新运作方法抢抓机遇精心谋划统筹推进重点突破奋发有为。大力推行"三具两基一抓手"的工作方法坚持超常规、不违规以领导方式和工作作风转变加快发展方式转变、提高办事效率。围绕贯彻落实省政府《关于支持南阳市经济社会加快发展的若干意见》加强学习研究搞好对省衔接尽快在相关专项规划编制、政策实施、项目安排、体制机制创新等方面取得实质性进展。改进目标管理细化分解目标层层落实责任加大督查力度强化考评问责确保各项目标任务落到实处。

各位代表!站在新起点实现新跨越责任重大使命光荣。让我们在省委、省政府和市委的坚强领导下齐心协力锐意进取开拓创新扎实苦干努力实现"十二五"良好开局为建设富强美好和谐新南阳而努力奋斗!
